# Delays in Seeking Medical Services in Elderly Patients With Senile Cataract

**DOI:** 10.3389/fpsyg.2022.930726

**Published:** 2022-07-12

**Authors:** Yifan Xiang, Haofeng Jiang, Lanqin Zhao, Qiong Liu, Haotian Lin

**Affiliations:** ^1^State Key Laboratory of Ophthalmology, Zhongshan Ophthalmic Center, Sun Yat-sen University, Guangzhou, China; ^2^Center for Precision Medicine, Sun Yat-sen University, Guangzhou, China

**Keywords:** senile cataract, medical delays, self-neglect, senile psychology, older patient

## Abstract

Delay in seeking medical services is common in elderly populations, which leads to disease progression and life difficulty. This study aims to assess the prevalence of delay in medical visits and treatment and define associated effects and factors in patients with senile cataract, which may help obtain a better understanding of late-life psychopathology and provide the basis for interventions. Patients aged more than 60 years were prospectively recruited in Zhongshan Ophthalmic Center (ZOC). All participants were diagnosed with binocular senile cataract and decided to have primary surgery in ZOC. The distributions of the popularity of delaying outpatient visits and treatment, the degrees of visual impairment, the influences on quality of life, and the reasons for delaying treatment among participants were accessed by the descriptive statistics. Factors associated with the perceptions of cataract treatment were accessed using a binary logistic regression model. A total of 400 senile patients aged from 60 to 94 years were enrolled. At diagnosis, 82 (20.5%) participants had a low vision with monocular acuity of both eyes below 0.05. All participants have felt that their normal lives were affected, and 64 (16%) participants felt that their lives were affected severely. Only 17 (4.25%) participants have sought for medical services immediately after feeling vision loss, and 294 (73.50%) participants have felt vision loss since a year ago before seeking medical help. A total of 298 (74.50%) participants have delayed the surgery time, and 229 (57.25%) patients delayed it for more than 12 months. There were 147 (36.75%) participants delaying surgery on account of no knowledge about it and 114 (28.50%) participants delaying surgery because of fear. There are a high proportion of elderly patients with senile cataract delaying their outpatient visits and surgery treatment, whose normal lives were severely affected. Increasing medical service propaganda about cataract and other common diseases in elderly populations would probably be helpful for improving perceptions of diseases and decreasing medical delays. Public needs to draw more attention to the healthy and medical status of the elderly ocular patients.

## Introduction

An increasingly remarkable population of senior citizens are benefiting from longevity with the advances in medical technology and health policy (Rousson and Paccaud, 2010; Zeng et al., 2017). Epidemiological studies show that 11% of the world’s population is over 60 years of age, and this proportion is expected to increase to 22% by 2050. However, this longevity presents new challenges for healthcare and society, as elderly people have higher morbidities of cancer and chronic diseases due to senescence (Fries, 1980; Gil and Withers, 2016). Therefore, early diagnosis and treatment are necessary and significant to allow for earlier recovery, better prognosis, and reduced medical expenditure (Bousquet et al., 2013; Davis et al., 2013).

Nonetheless, delayed medical visits and treatment are a common and serious problem in elderly patients (Mosqueda and Dong, 2011). Physicians have a high frequency of encounters with elderly patients who neglect symptoms of diseases until their lives are severely affected, which likely leads to longer treatment, more complications, and worse prognosis (Schoepfer et al., 2013; de Haan et al., 2018). As a consequence, delaying treatment provokes disease progression and life deterioration (Yurdakul et al., 2015). In addition to critically impacting patients’ lives, delaying treatment may cause enormous economic and mental pressure on family members, as well as a tremendous waste of healthcare resources, which negatively affects the aging population (Pita Fernández et al., 2010; Li et al., 2018).

Senile cataract is the most common eye disease in elderly adults; it has a prevalence of 13–50% among people older than 60 years worldwide and causes vision loss, sometimes even blindness (Liu et al., 2017; Song et al., 2018). Senile cataract progresses slowly and regularly leads to the deterioration of visual function and life quality, similar to many perennial older diseases. Surgery is the only effective treatment for cataract (Lam et al., 2015; Liu et al., 2017). However, the rate of surgery for cataract is low, and a large proportion of elderly patients with cataract miss early diagnosis and treatment and come to hospitals with low vision and a reduced quality of life (Hashemi et al., 2014; Wang et al., 2016; Pawiroredjo et al., 2017).

Currently, great efforts have been made in biomedical research regarding the pathways of anti-aging (Fontana et al., 2014). Nevertheless, less attention has been paid to the actuality of the delay in diagnosis and treatment in elderly patients. Our study investigated elderly patients with senile cataract to obtain more specific knowledge about the effects of delaying diagnosis and treatment. We analyzed the prevalence, effect, and risk factors of delays in seeking medical help and proposed some solutions to improve the current situation.

## Materials and Methods

A prospective study was carried out at the Zhongshan Ophthalmic Center (ZOC), Guangdong, China, from June 2017 to July 2018. A total of 400 patients who were diagnosed with senile cataract and planned to undergo cataract surgery were consecutively recruited. This study was approved by the Ethical Review Committee of ZOC. The tenets of the Declaration of Helsinki were followed throughout this study.

### Sample Selection

The inclusion criteria in the study were as follows: (1) diagnosed with senile cataract at the ZOC; (2) aged more than 60 years; (3) planning to undergo cataract surgery for the first time; (4) from Guangzhou city; and (5) signed the informed consent document. The exclusion criteria were as follows: (1) diagnosed with other ocular diseases and (2) diagnosed with cognitive impairment. Patients meeting the included requirements were enrolled consecutively.

### Data Collection and Interpretation

The collected data included Snellen acuity, demographic information, visual function, and quality of life. All the data for the same person were obtained on the same day. The acuity data were obtained from clinical test results. The demographic information was collected with a questionnaire and included sex, age, education, economic conditions, awareness of senile cataract, family support status, and so on. The degree of visual impairment and effects on quality of life were collected using a scale of vision function and quality of life (Fan et al., 2008). The demographic information questionnaire was designed and revised based on suggestions from eight professors in the cataract department and 20 patients with senile cataract seen at the ZOC (refer to Supplementary Material). The scales were finished under instructions, and the answers to each question were recorded.

To facilitate the analysis, the results of visual impairment and effects on quality of life were transformed into numerical values ranging from 0 to 100. In each question, there are four options representing four degrees of severity; in the analysis, we adopted values of 1, 2, 3, and 4 to represent the four options. For questions 7a, 7b and 11a, 11b on the visual function scale, only the answer with the greater value was used in the analysis. Each participant’s final score was divided by the total scale score and then multiplied by 100 to obtain a result ranging from 0 to 100. The greater this value was, the more severe the patient’s visual impairment and effects on quality of life were.

### Statistical Analysis

Patients going to outpatient visits more than 1 week after vision loss and patients deciding to accept cataract surgery more than 1 week after the doctor’s recommended time for surgery were defined as delays in medical visits and treatments in the analysis. We described the distributions of the acuity level, demographic characteristics, visual impairment, and effects on quality of life for different age groups and the duration of delays in medical visits. Then, we explored the factors associated with the perceptions of drug and surgical treatment for cataract. All analyses were performed using IBM SPSS 22.0.

We performed descriptive statistics to compare the distributions of sex, education level, income, acuity, and different durations of delay in medical visits and treatment among individuals of different ages. We then determined the descriptive statistics of individuals with different durations to identify significant differences using the chi-square tests or using Fisher’s exact test if there is an expected frequency of less than 5. A binary logistic regression model was applied to explore the factors associated with the perceptions of drug and surgical treatment. Exp (B) values signify the influence degrees of associated factors; values greater than 1 indicate a positive effect, and values less than 1 indicate a negative effect. Fisher’s exact test was conducted to analyze the distributions regarding reasons for delaying surgery treatment and reasons for finally undergoing surgery. *P*-value < 0.05 was considered statistically significant.

## Results

A total of 400 patients were enrolled. Participants aged 60–94 years were categorized into different age groups according to sex, education level, income, visual acuity, and different durations of delays in medical visits and treatment (refer to Table 1). Four participants were older than 90 years. A total of 154 (38.5%) participants were male, and 92% had a high school education or less. The monocular Snellen acuity of participants ranged from no light perception to 0.3, and 82 (20.5%) participants had blindness, with monocular acuity of both eyes below 0.05. In addition, the education level exhibited distributional differences among different age groups. The younger group has a higher education level. The duration of delay in cataract surgery was associated with age, and the older patients tended to delay cataract surgery longer.

**TABLE 1:** Demographic characteristics of the participants according to age.

	Age (year)	60–69	70–79	≥80	*P*-Value
Sex	Male	44 (44.00%)	62 (35.63%)	48 (38.10%)	0.389
	Female	56 (56.00%)	112 (64.37%)	78 (61.90%)	
Education	Primary school and below	46 (46.00%)	97 (55.75%)	87 (69.05%)	0.001
	High school diploma	49 (49.99%)	60 (34.48%)	29 (23.02%)	
	Bachelor’s degree and above	5 (5.00%)	17 (9.77%)	10 (7.94%)	
Annual personal income (yuan)	0–30,000	47 (47.77%)	59 (33.91%)	43 (34.13%)	0.163
	30,000–50,000	31 (31.00%)	77 (44.25%)	55 (43.65%)	
	>50,000	22 (22.00%)	38 (21.84%)	28 (22.22%)	
Best monocular acuity (Snellen acuity)	0–0.05	22 (22.00%)	38 (21.84%)	22 (17.46%)	0.180
	0.06–0.20	50 (50.00%)	83 (47.70%)	51 (40.48%)	
	0.21–0.3	28 (28.00%)	53 (30.46%)	53 (42.06%)	
Delay in outpatient visits	No*^a^*	4 (4.00%)	8 (4.60%)	5 (3.97%)	0.276
	0–6 months	5 (5.00%)	22 (12.64%)	11 (8.73%)	
	7–12 months	18 (18.00%)	17 (9.77%)	16 (12.70%)	
	>12 months	73 (73.00%)	127 (72.99%)	94 (74.60%)	
Delay in cataract surgery	No*^b^*	34 (34.00%)	43 (24.71%)	25 (19.84%)	0.031
	0–6 months	8 (8.00%)	21 (12.07%)	10 (7.93%)	
	7–12 months	11 (11.00%)	7 (4.02%)	12 (9.52%)	
	>12 months	47 (47.00%)	103 (59.20%)	79 (62.70%)	

*Participants aged 60–94 years were categorized into different age groups according to sex, education level, income, visual acuity, and different durations of delay in medical visits and treatment.*

*^a^Patients go to outpatient visits within 1 week after feeling the vision loss.*

*^b^Patients decide to accept cataract within 1 week after the doctor’s recommended time for surgery.*

The distributions of sex, age, education level, cohabitation status, income, visual impairment, and effects on quality of life of participants with different durations of delay in treatment are presented in Table 2. There was no significant distributional difference among the three groups. A total of 294 (73.50%) participants had experienced vision loss for a year before seeking medical help at the hospital. Only 55 (13.75%) participants had come to the hospital within 6 months of noticing vision impairment. Notably, 232 (58.00%) participants felt that their normal lives were mildly affected, and 64 (16.00%) participants felt that their lives were severely affected. Also, 46 (11.5%) participants lived alone. None of the patients lived in a retirement home. The visual impairment evaluation scores ranged from 47 to 100. Individuals with scores ranging from 40 to 59 accounted for more than half the participants, and those with a high level of visual impairment (scores between 80 and 100) constituted 14.25% of all participants. The effects on quality of life scores ranged from 31 to 100. In total, 47% of participants had scores ranging from 50 to 69, and 34.25% indicated a high level of effects on quality of life (scores between 70 and 100).

**TABLE 2:** Demographic characteristics, visual function, and life quality of participants according to the duration of delay in medical visits after feeling vision loss.

		No	0–6 months	6–12 months	>12 months	*P*-Value
Total		17	38	51	294	
Sex	Male	5 (29.4%)	18 (47.4%)	16 (31.4%)	115 (39.1%)	0.392
	Female	12 (70.6%)	20 (52.6%)	35 (68.6%)	179 (60.9%)	
Age	60–69 years old	4 (23.5%)	5 (13.2%)	18 (35.3%)	73 (24.8%)	0.276
	79–79 years old	8 (47.1%)	22 (57.9%)	17 (33.3%)	127 (43.2%)	
	>80 years old	5 (29.4%)	11 (28.9%)	16 (31.4%)	94 (32.0%)	
Education	Primary school degree and below	14 (82.4%)	20 (52.6%)	26 (51.0%)	170 (57.8%)	0.403
	High school degree	3 (17.6%)	14 (36.8%)	21 (41.2%)	100 (34.0%)	
	Bachelor degree and above	0	4 (10.5%)	4 (7.8%)	24 (8.2%)	
Feel life influenced	Mildly	10 (58.8%)	26 (68.4%)	25 (49.0%)	171 (58.2%)	0.498
	Moderately	3 (17.6%)	9 (23.7%)	15 (29.4%)	77 (26.2%)	
	Severely	4 (23.5%)	3 (7.9%)	11 (21.6%)	46 (15.6%)	
Live with	None	2 (11.8%)	6 (15.8%)	2 (3.9%)	36 (12.2%)	0.144
	Spouse	6 (35.3%)	12 (31.6%)	13 (25.5%)	66 (22.4%)	
	Children	5 (29.4%)	10 (26.3%)	22 (43.1%)	77 (26.2%)	
	Spouse and children	4 (23.5%)	10 (26.3%)	14 (27.5%)	115 (39.1%)	
Annual personal income	0–30,000	6 (35.3%)	17 (44.7%)	20 (39.22%)	106 (36.1%)	0.670
	30,000–50,000	9 (52.9%)	16 (42.1%)	20 (39.2%)	118 (40.1%)	
	>50,000	2 (11.8%)	5 (13.2%)	11 (21.6%)	70 (23.8%)	
Best monocular acuity (Snellen acuity)	0.00–0.05	5 (29.4%)	3 (7.9%)	13 (25.5%)	51 (17.3%)	0.249
	0.06–0.20	9 (52.9%)	23 (60.5%)	22 (43.1%)	164 (56.1%)	
	0.21–0.30	3 (17.6%)	12 (31.6%)	16 (31.4%)	79 (26.5%)	
Evaluation of visual impairment	40–59	5 (29.4%)	20 (52.6%)	25 (49.0%)	163 (55.4%)	0.154
	60–79	9 (52.9%)	16 (42.1%)	19 (37.3%)	86 (29.3%)	
	80–100	3 (17.6%)	2 (5.3%)	7 (13.7%)	45 (15.3%)	
Evaluation of the effects on quality of life	30–49	3 (17.6%)	7 (18.4%)	12 (23.5%)	53 (18.0%)	0.664
	50–69	5 (29.4%)	19 (50.09%)	24 (47.1%)	140 (47.6%)	
	70–100	9 (52.9%)	12 (31.6%)	15 (29.4%)	101 (34.4%)	

*The distribution of sex, age, education level, cohabitation status, income, visual impairment, and effects on the quality of life of participants with different durations of delay in treatment are presented.*

**FIGURE 1:**
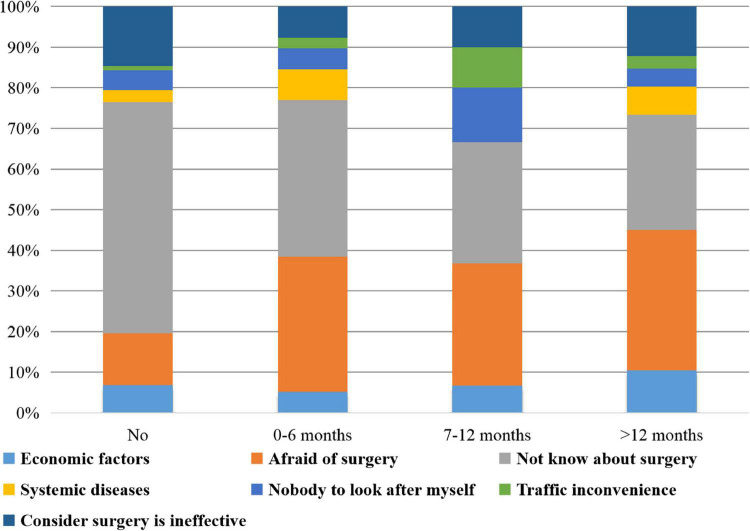
Distribution of durations of delaying surgery treatment and reasons for it. A total of 298 (74.50%) participants delayed surgery, and 229 (57.25%) delayed it for more than 12 months. The reasons why patients delayed surgery exhibited distributional differences among four groups (*P* < 0.001). There were 114 (28.50%) participants delaying surgery on account of fear and 147 (36.75%) delaying it because of having no knowledge about it.

**FIGURE 2:**
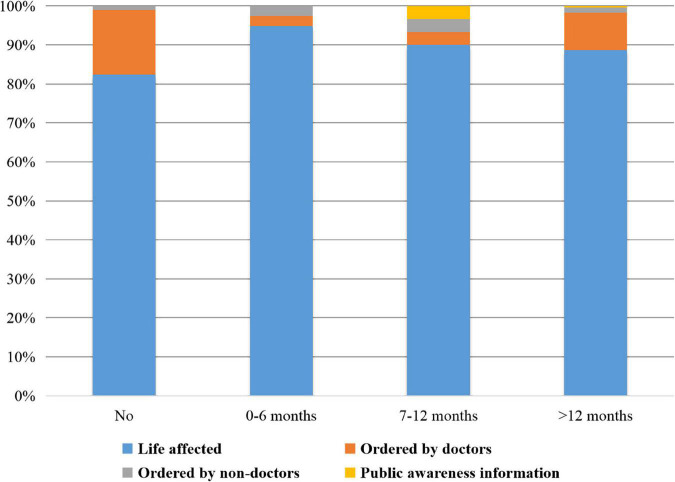
Distribution of durations of delaying surgery and reasons for finally undergoing surgery. The reasons why patients finally accepted surgery indicated no distributional differences among the four groups (*P* = 0.066). Notably, 351 (87.75%) patients chose to undergo surgery because their lives were affected and 41 (10.25%) patients chose to undergo surgery as recommended by doctors.

The distributions of the durations of delays in surgery and the reasons for the final surgery choice are presented in Figure 1. A total of 102 patients chose to have surgery according to the doctors’ orders. However, 298 (74.50%) participants delayed the surgery time, and 229 (57.25%) patients delayed it for more than 12 months beyond the doctor’s recommended time. The reasons why patients delayed surgery exhibited distributional differences among four groups (*P* < 0.001). The reasons patients cited for finally undergoing surgery were multiple; the most common reason was that their lives were being affected by the cataract, which was selected by 351 (87.75%) patients (shown in Figure 2). A total of 41 (10.25%) patients chose to undergo surgery as recommended by doctors. The reasons why patients finally accepted surgery indicated no distributional differences among the four groups (*P* = 0.066).

The participants held different opinions about the effectiveness of drug treatment for senile cataract. Notably, 56 participants thought that the drug treatment was valid for cataract, 188 held the opposite belief, and 156 had no opinion about the drug treatment. The factors associated with perceptions of drug treatment are shown in Table 3. Several factors were associated with the participants’ perceptions of drug treatment, including effects on quality of life, attempts at drug therapy, and the frequencies of required physical tests. Attempted drug therapy had the strongest relationship with the participants’ perceptions of drug therapy. The participants whose doctors had recommended were most likely to consider the drug treatment effective. Patients who had not undergone physical tests tended to hold the same opinion.

**TABLE 3:** Factors associated with perceptions of drug treatment.

	Exp (B)	Sig.	95% CI
**Effects on quality of life**			
No	1.000		
Yes	4.345	0.007	1.502–12.571
**Drug treatment**			
None	1.000		
Recommended by doctors	18.570	0.000	5.902–58.428
Recommended by self	1.155	0.730	0.508–2.625
**Physical test in 3 years**			
None	1.000		
When feeling unwell	0.690	0.545	0.208–2.294
Annually	0.169	0.002	0.055–0.520

*Participants who thought drug treatment was invalid were considered the standard group, and those who believed that drug treatment was effective were the comparison group. Exp (B) signifies the degree of influence on perceptions of drug therapy, with a value greater than 1 indicating a positive effect.*

The elderly patients had different perceptions regarding cataract surgery. There were 147 participants who had no idea about the surgery treatment. Four factors were associated with awareness of cataract surgery (Table 4). Patients who delayed outpatient visits and were mildly affected tended not to know about surgical treatment. In addition, patients who held the opinion that senility causes vision loss were less likely to know about surgical treatment.

**TABLE 4:** Factors associated with perceptions of surgical treatment.

	Exp (B)	Sig.	95% CI
**Delayed outpatient visits**			
<6 month	1.000		
6–12 months	2.254	0.066	0.948–5.356
>12 months	2.184	0.016	1.156–4.124
**Reason for vision loss**			
Senility	1.000		
Cataract	3.721	0.000	2.308–5.999
Other	1.131	0.773	0.491–2.604
**Effects on quality of life**			
Severe	1.000		
Moderate	0.303	0.004	0.136–0.676
Mild	0.269	0.001	0.128–0.566
**Physical test in 3 years**			
None	1.000		
When feeling unwell	0.387	0.001	0.219–0.685
Annually	0.840	0.757	0.277–2.544

*Participants who thought the surgical treatment was invalid were considered the standard group, and those who believed that surgical treatment was effective were the comparison group. Exp (B) signifies the degree of influence on awareness of cataract surgery, with a value greater than 1 indicating a positive effect.*

## Discussion

Some surveys have reported the status of and factors associated with delayed treatment in patients with different diseases, especially cancers and infectious diseases (Saver et al., 2016; Getnet et al., 2017; de Haan et al., 2018; Leung et al., 2018; Vizza et al., 2018). However, the status of delayed diagnosis and treatment in elderly patients has not received much attention and warrants greater concern. A large proportion of elderly patients with senile cataract who were recruited for our study delayed their outpatient visits and surgical treatment. We describe the prevalence of delayed outpatient visits and treatment among elderly patients with senile cataract and analyze the factors associated with disease perceptions.

A high proportion of the elderly patients (95.75%) in our study delayed their outpatient visits and diagnosis, and 74.5% delayed surgical treatment. It has been reported that self-neglect is the most common form of elder mistreatment; it usually manifests as neglecting self-health and is reportedly on the rise (Payne and Gainey, 2005). Self-neglect is a public health problem that crosses all demographic and socioeconomic strata of the aging population (Mosqueda and Dong, 2011). Similar to previous studies, our study revealed a relationship between age and delaying treatment, with older patients tending to have longer durations of treatment delays (Knoepfli et al., 2019). We suppose that older patients are more likely to confuse senility and disease symptoms and have fewer perceptions regarding disease treatment.

The senile cataract progresses slowly, gradually affecting normal life (Lam et al., 2015). Similar to many older diseases, cataract develops chronically and is not easily perceived by elderly people (Steel, 2005). In our study, 37% of participants believed that senility causes vision loss. The degradation of body functions and symptoms of chronic diseases are frequently confused by elderly populations (Tchkonia and Kirkland, 2018). As a consequence, a great proportion of elderly patients neglect early symptoms and miss opportunities for early diagnosis and treatment. We propose that elderly patients with senile cataract are also likely to miss opportunities for early diagnosis and treatment. In addition, incorrect perceptions of disease treatment can lead to delayed treatment; indeed, 147 (36.75%) participants have no thoughts regarding surgical treatment, the only effective therapy for senile cataract. Increased information about common diseases in elderly populations would probably be helpful for improving perceptions of diseases. More promotional brochures and medical lectures can be delivered at senior citizens’ activity centers.

The visual impairment and effects on quality of life experienced by elderly patients who do not receive early cataract treatment can seriously affect their normal life. The participants’ visual impairment and effects on their quality of life scale scores indicated that their normal life was greatly affected. In addition, although some of them lived with spouses and children, their family members probably did not notice their declining life functions or advise them to go to hospitals, as more than 80% of the participants finally sought treatment because of the effects on quality of life rather than because of recommendations from family members. The attention paid to elderly populations by their family members is limited. It has been reported that perceived neglect and reduced care were associated with increased mortality risk in a general population of elderly adults (Barnes et al., 2008). The health status of elderly family members should receive greater attention from the younger family members who are advised to learn about common older diseases and their early symptoms. Younger individuals have the responsibility to care for the health status of the elderly and suggest them to go to the hospital when something is wrong (Hudson and Johnson, 1986).

It is important to identify modifiable factors for the improvement of perceptions of cataract treatment. The factors associated with the perceptions of cataract treatment are presented in Tables 3, 4. The factor “effects on quality of life” is an important cause associated with perceptions of drug treatment. Patients who have difficulty in daily life may have more intrinsic motivation to find an effective way to solve the cataract problem. The doctors’ recommendations have an obvious effect on the incorrect beliefs about treatment, which illustrates the dominant role of doctors in elderly patients’ disease awareness. In addition, frequent physical tests have a negative effect on perceptions regarding cataract treatment. People who undergo regular physical tests usually take their personal health more seriously; however, their perceptions of diseases are mostly incorrect, which is beyond our expectations. We presume that conservative treatment approaches from doctors based on test results in the early stage of cataract likely lead to improper perceptions of disease treatment among patients. Patients who think that the reason for vision loss is cataract instead of senility and feel severely affected have better perceptions of surgical treatment, which is understandable. The dissemination of knowledge of cataract disease may contribute to early disease treatment. Similar to previous results, modifiable social and behavioral factors can enhance the perceptions of diseases and consequently improve the prognosis of older people, reinforcing the evidence for establishing intervention strategies (Fried et al., 2004; Glymour and Osypuk, 2012). More detailed treatment information after diagnosis, including the recommended treatment regimens for different stages of the disease, recommended follow-up intervals, and the consequences of disease progression, need to be provided to elderly patients *via* doctor–patient communication, medical examination reports, and so on.

There were limitations to this study. First, the participants were from Guangzhou city, and only patients with senile cataract were enrolled. Caution should be used when generalizing the results to other populations. Second, this was a cross-sectional survey that did not allow cause–effect relationships to be determined. Longitudinal studies should be considered in the future. In addition, more studies are warranted to investigate the prevalence of and factors associated with delays in medical visits and treatment in elderly patients for other severe and acute diseases.

## Conclusion

Our study is the first to illuminate the prevalence of and effect of delays in medical visits and treatment in elderly patients with senile cataract and to draw attention to the health and medical status of the elderly. Some interventions are necessary to improve disease knowledge and address self-neglect in elderly patients. We propose that younger individuals attach more importance to the health of elderly family members.

## Data Availability Statement

The raw data supporting the conclusions of this article will be made available by the authors, without undue reservation.

## Ethics Statement

The studies involving human participants were reviewed and approved by Ethical Review Committee of ZOC. The patients/participants provided their written informed consent to participate in this study.

## Author Contributions

YX and HL contributed to the conception and design of the study. HJ contributed to the acquisition of data. LZ and QL performed the statistical analyses. YX contributed to drafting the manuscript. HL contributed to revising the manuscript critically for important intellectual content. All authors contributed to the interpretation of data and approved the final version of this manuscript.

## Conflict of Interest

The authors declare that the research was conducted in the absence of any commercial or financial relationships that could be construed as a potential conflict of interest.

## Publisher’s Note

All claims expressed in this article are solely those of the authors and do not necessarily represent those of their affiliated organizations, or those of the publisher, the editors and the reviewers. Any product that may be evaluated in this article, or claim that may be made by its manufacturer, is not guaranteed or endorsed by the publisher.
