# Supplementary material for: Delays in Seeking Medical Services in Elderly Patients With Senile Cataract
Source: Front Psychol. 2022 Jul 12;13:930726. doi: 10.3389/fpsyg.2022.930726 (PMC9314650; doi:10.3389/fpsyg.2022.930726)
Supplement: Supplementary file 1 [file Data_Sheet_1.PDF]

The questionnaire for demographic information collection

Name: Age:

Sex: ①Male ②Female

1 How long you felt the vision loss before going to hospitals and seeking medical help?

① Less than 1 week;

② 1 week-6 months;

③ 7-12 months;

④ more than 12 months

2 What do you think is the cause of vision loss?

① Senility;

② Cataract;

③ Other;

3 How long has it been since doctor’s recommended time for surgery?

① Less than 1 week;

② 1 week-6 months;

③ 7-12 months;

④ more than 12 months

4 What are your main activities?

① Work;

② Taking care of the family members;

③ Other;

5 Do you think your daily life has been affected?

① Severely affected;

② Moderately affected;

③ Mildly affected;

④ Not affected;

6 Have you ever received any cataract medication?

① No;

② Ordered by doctors;

③ Ordered on self;

④ Other;

7 What do you think of the effect of drug therapy on cataract?

① Effective;

② Not effective;

③ No idea;

8 Why did you choose to have surgery now?

① Economic limit;

② Systemic diseases;

③ Afraid of surgery;

④ Have no idea about surgery;

⑤ Nobody to look after myself;

⑥ Traffic inconvenience;

⑦ Surgical ineffective;

⑧ Other;

9 What do you think led you to choose cataract surgery finally?

① Life affected;

② Ordered by doctors;

③ Ordered by non-doctors;

④ Medical publicity;

⑤ Other;

10 Have you had physical examinations in the past 3 years?

① No

② When feel unwell

③ Regular test

11 What is your annual income?

① 0-10,000 yuan

② 10,000-30,000 yuan

③ 30,000-50,000

④ >50,000 yuan

12 Who do you live with? (multiple choice)

① No

② Spouse

③ Children

④ Friends in retirement home

13 What is your educational degree?

① Primary school degree and below

② Junior high school degree

③ Senior high school degree

④ Bachelor degree and above

14 What is your job?

① No job

② Teacher

③ Worker

④ Farmer

⑤ Civil servant

⑥ Businessman

⑦ Other
